# Supplementary material for: 24-hour time-use behaviors and maternal depressive symptoms during early pregnancy: Compositional data analysis
Source: medRxiv. 2026 Nov 1:2026.10.29.26366835. Preprint. [Version 1] doi: 10.64898/2026.10.29.26366835 (PMC13734214; doi:10.64898/2026.10.29.26366835)
Supplement: Supplementary file 1 [file media-1.pdf]

24-hour time-use behaviors and maternal depressive symptoms during early pregnancy: Compositional data analysis

Authors:

Takashi Yoshimasu, MD, MPH TAY73@pitt.edu

Namhyun Kim, PharmD, MS NAK264@pitt.edu

James Matuk, PhD JAM925@pitt.edu

Christina M. Personette, BS CMP309@pitt.edu

Reagan E. Garcia, PhD, MPH REM168@pitt.edu

Michele D. Levine, PhD LEVINEM@upmc.edu

Marquis Hawkins, PhD MAH400@pitt.edu

Appendix

Appendix 1. Description of g-computation procedures

Let

$$\boldsymbol{X} = (X_1, X_2, X_3, X_4)^T$$

denote 24-h time-use behavior components, where

- X_1: Moderate-to-Vigorous Intensity Physical Activity (MVPA; min/day)
- X_2: Light intensity Physical Activity (LPA; min/day)
- X_3: Inactivity (IA; min/day)
- X_4: Sleep (min/day)

These components are subject to the constraint $\sum_{k=1}^{4} X_k = T$. The isometric log-ratio (ILR) transformation $\boldsymbol{Z} = ilr(\boldsymbol{X})$ is defined component-wise as

$$Z_j = \sqrt{\frac{4-j}{4-j+1}} \, ln\left(\frac{X_j}{\left(\prod_{k=j+1}^{4} X_k\right)^{1/(4-j)}}\right), j = 1, 2, 3.$$

Counterfactual exposure levels were defined as modifications of each individual's observed compositional vector $\boldsymbol{x}_i^{obs}$. Specifically, for a component x_{ih}^{obs}($h \in \{1,2,3,4\}$; the component to be manipulated), we considered a relative change of r_{ih}, i.e., $a = (1 + r_{ih})\, x_{ih}^{obs}$ (a $100r\%$ change relative to the individual's observed value), while the remaining components were rescaled proportionally so that their relative composition was preserved and the total remained T. To satisfy the compositional constraint, a relative change by a factor of $1 + r_{ih}$ necessitates a relative change in other components by a factor of $1 - s_{ih}$, where $1 - s_{ih} = 1 - r_{ih} \frac{x_h^{obs}}{1-x_h^{obs}} = \frac{1-a}{1-x_h^{obs}}$ [1]. Therefore, a counterfactual component vector of each individual $\boldsymbol{x}_i^*$ with $x_{ih}^* = a$ is defined as

$$\boldsymbol{x}_i^* = \boldsymbol{g}_{h,a}\left(\boldsymbol{x}_i^{obs}\right)$$

where

$$\left[\boldsymbol{g}_{h,a}(\boldsymbol{x})\right]_k = \begin{cases} a, & k = h, \\ x_k \dfrac{T-a}{T-x_h^{obs}}, & k \neq h. \end{cases}$$

k indexes the components of the vector ($k \in \{1,2,3,4\}$)). Therefore, $\boldsymbol{z}^* = ilr\left(\boldsymbol{g}_{h,a}(\boldsymbol{x})\right)$ is the ILR representation of counterfactual values where $x_h = a$. This implies that counterfactual exposure value $\boldsymbol{z}^*$ is a function of the observed vector of time-use components and the counterfactual value of a.

Assuming positivity, consistency, and conditional exchangeability given covariates, our target estimand is defined as

$$
\begin{aligned}
E[Y^{X_h=a}] &= E_{\boldsymbol{X},\boldsymbol{C}}\left[E\left[Y^{\boldsymbol{g}_{h,a}(\boldsymbol{x})} \mid \boldsymbol{X} = \boldsymbol{x}, \boldsymbol{C} = \boldsymbol{c}\right]\right] \\
&= E_{\boldsymbol{X},\boldsymbol{C}}\left[E\left[Y \mid \boldsymbol{X} = \boldsymbol{g}_{h,a}(\boldsymbol{x}), \boldsymbol{C} = \boldsymbol{c}\right]\right] \\
&= E_{\boldsymbol{X},\boldsymbol{C}}\left[E[Y \mid \boldsymbol{Z} = \boldsymbol{z}^{*}, \boldsymbol{C} = \boldsymbol{c}]\right].
\end{aligned}
$$

where Y denotes the Edinburgh Postnatal Depression Scale (EPDS) total score, and $\boldsymbol{C}$ denotes covariates. The forth equality in the estimand definition holds since the ILR transformation is a bijective map as long as the compositional time-use vector has all strictly positive elements.

The inner expectation in the target estimand is specified through a regression model on the log scale

$$\log(Y) = \beta_0 + \boldsymbol{Z}^{\boldsymbol{T}}\boldsymbol{\beta} + \boldsymbol{C}^{\boldsymbol{T}}\boldsymbol{\gamma} + \epsilon,$$

with $\epsilon \sim (0, \sigma^2)$ and $E[exp(2\epsilon)] < \infty$. The conditional expectation on the EPDS scale is given through Duan's Smearing Method [2,3], $E[Y \mid \boldsymbol{Z} = \boldsymbol{z}_{ha}^{*}, \boldsymbol{C} = \boldsymbol{c}] = \exp(\beta_0 + {\boldsymbol{z}_{ha}^{*}}^{\boldsymbol{T}}\boldsymbol{\beta} + \boldsymbol{c}^{\boldsymbol{T}}\boldsymbol{\gamma})\, E[\exp(\epsilon)]$. G-computation is used to estimate the target estimand, where predicted potential outcomes for each participant, i, is generated through

$$\hat{y}_i = \exp(\hat{\beta}_0 + \boldsymbol{z}_i^{*\boldsymbol{T}}\hat{\boldsymbol{\beta}} + \boldsymbol{c}_{\boldsymbol{i}}^{\boldsymbol{T}}\hat{\boldsymbol{\gamma}}) \times \frac{1}{n}\sum_{l=1}^{n}\exp(\hat{\epsilon}_l), i = 1, \dots, n,$$

where $\boldsymbol{z}_i^{*}$ is the ILR representation of individual's counterfactual vectors where $x_h = a$, and $\boldsymbol{c}_{\boldsymbol{i}}$ are the observed covariate values for the participant. Finally, $\hat{E}[Y^{X_h=a}] = \frac{1}{n}\sum_{i=1}^{n}\hat{y}_i$.

Appendix Table 1. Participant characteristics among the primary analysis and excluded

**Characteristic**	**Statistic**	**Main analysis** $N = 255$	**Excluded** $N = 57$
**Gestational week**	Median (IQR)	14 (11, 15)	15 (12, 16)
**Maternal age (years)**	Mean (SD)	30.5 (4.9)	30.3 (5.1)
**Body Mass Index (kg/m²)**	Median (IQR)	31.1 (28.1, 36.4)	31.7 (28.6, 35.0)

Characteristic	Statistic	Main analysis<br>*N* = 255	Excluded<br>*N* = 57
**Race/Ethnicity**	n (%)		
Non-Hispanic White		174 (68.2%)	33 (57.9%)
Non-Hispanic Black		53 (20.8%)	16 (28.1%)
Non-Hispanic Asian		1 (0.4%)	3 (5.3%)
Hispanic		12 (4.7%)	3 (5.3%)
Other identities		14 (5.5%)	2 (3.5%)
Missing		1 (0.4%)	0 (0.0%)
**Educational status**	n (%)		
High school		37 (14.5%)	10 (17.5%)
Some college		68 (26.7%)	16 (28.1%)
4-year college graduate		71 (27.8%)	15 (26.3%)
Graduate school		79 (31.0%)	16 (28.1%)
**Household income**	n (%)		
≤US$30,000		75 (29.4%)	22 (38.6%)
US$30,001—70,000		59 (23.1%)	10 (17.5%)
US$70,001—100,000		53 (20.8%)	12 (21.1%)
>US$100,000		68 (26.7%)	12 (21.1%)
Missing		0 (0.0%)	1 (1.8%)
**Insurance status**	n (%)		
Provided by employer		164 (64.3%)	33 (57.9%)
Medicare/Medicaid		85 (33.3%)	22 (38.6%)
Personal Policy		5 (2.0%)	1 (1.8%)
No Health Insurance		1 (0.4%)	0 (0.0%)
Missing		0 (0.0%)	1 (1.8%)
**Employment**	n (%)		
Working		196 (76.9%)	41 (71.9%)
Not working		59 (23.1%)	16 (28.1%)
**Cohabitation status**	n (%)		
Living with partner		210 (82.4%)	48 (84.2%)

Characteristic	Statistic	Main analysis *N* = 255	Excluded *N* = 57
Living without partner		45 (17.6%)	9 (15.8%)
**Study year**	n (%)		
2017-2019		174 (68.2%)	29 (50.9%)
2020-2022		81 (31.8%)	28 (49.1%)
**EPDS[1] score**	Median (IQR)	5.0 (2.0, 8.0)	4.0 (2.0, 9.0)

[1]EPDS, Edinburgh Postnatal Depression Scale.

Appendix Table 2. The estimated change by 30 min reallocation

Behavior gaining time[1-3]	Behavior losing time	Estimated EPDS[3] scores (95% CI)
MVPA	LPA	-0.01 (-0.39, +0.38)
MVPA	IA	-0.11 (-0.36, +0.13)
MVPA	Sleep	-0.11 (-0.37, +0.14)
LPA	MVPA	+0.06 (-0.40, +0.63)
LPA	IA	-0.09 (-0.25, +0.08)
LPA	Sleep	-0.10 (-0.25, +0.06)
IA	MVPA	+0.15 (-0.20, +0.59)
IA	LPA	+0.10 (-0.08, +0.29)
IA	Sleep	-0.01 (-0.11, +0.08)
Sleep	MVPA	+0.16 (-0.21, +0.62)
Sleep	LPA	+0.10 (-0.06, +0.28)
Sleep	IA	+0.00 (-0.08, +0.11)

[1]MVPA, moderate-to-vigorous physical activity; [2]LPA, light physical activity; [3]IA, inactivity; [4]EPDS, Edinburgh Postnatal Depression Scale.

Appendix Table 3. The estimated change by 60 min reallocation

Behavior gaining time[1-3]	Behavior losing time	Estimated EPDS[3] scores (95% CI)
MVPA	LPA	+0.01 (-0.66, +0.78)
MVPA	IA	-0.19 (-0.62, +0.23)

Behavior gaining time[1-3]	Behavior losing time	Estimated EPDS[3] scores (95% CI)
MVPA	Sleep	-0.20 (-0.64, +0.25)
LPA	MVPA	+0.27 (-0.94, +2.19)
LPA	IA	-0.18 (-0.48, +0.14)
LPA	Sleep	-0.19 (-0.47, +0.11)
IA	MVPA	+0.46 (-0.63, +2.09)
IA	LPA	+0.21 (-0.17, +0.62)
IA	Sleep	-0.01 (-0.22, +0.15)
Sleep	MVPA	+0.47 (-0.66, +2.18)
Sleep	LPA	+0.22 (-0.13, +0.61)
Sleep	IA	+0.00 (-0.16, +0.22)

[1]MVPA, moderate-to-vigorous physical activity; [2]LPA, light physical activity; [3]IA, inactivity; [4]EPDS, Edinburgh Postnatal Depression Scale.

Appendix Table 4. Maternal demographics among the primary analysis and sensitivity analysis

Characteristic	Statistic	Overall *N* = 255	Sensitivity analysis *N* = 192	Excluded *N* = 63
**Gestational week**	Median (IQR)	14 (11, 15)	14 (10, 15)	14 (12, 15)
**Maternal age (years)**	Mean (SD)	30.5 (4.9)	30.6 (5.0)	30.4 (4.7)
**Body Mass Index (kg/m²)**	Median (IQR)	31.1 (28.1, 36.4)	30.7 (28.0, 35.6)	32.7 (28.6, 37.9)
**Race/Ethnicity**	n (%)			
Non-Hispanic White		174 (68.2%)	139 (72.4%)	35 (55.6%)
Non-Hispanic Black		53 (20.8%)	33 (17.2%)	20 (31.7%)
Non-Hispanic Asian		1 (0.4%)	0 (0.0%)	1 (1.6%)
Hispanic		12 (4.7%)	7 (3.6%)	5 (7.9%)
Other identities		14 (5.5%)	12 (6.3%)	2 (3.2%)
Missing		1 (0.4%)	1 (0.5%)	0 (0.0%)
**Educational status**	n (%)			
High school		37 (14.5%)	26 (13.5%)	11 (17.5%)

Characteristic	Statistic	Overall<br>*N* = 255	Sensitivity analysis<br>*N* = 192	Excluded<br>*N* = 63
Some college		68 (26.7%)	45 (23.4%)	23 (36.5%)
4-year college graduate		71 (27.8%)	56 (29.2%)	15 (23.8%)
Graduate school		79 (31.0%)	65 (33.9%)	14 (22.2%)
**Household income**	n (%)			
≤US$30,000		75 (29.4%)	51 (26.6%)	24 (38.1%)
US$30,001—70,000		59 (23.1%)	41 (21.4%)	18 (28.6%)
US$70,001—100,000		53 (20.8%)	42 (21.9%)	11 (17.5%)
>US$100,000		68 (26.7%)	58 (30.2%)	10 (15.9%)
**Insurance status**	n (%)			
Provided by employer		164 (64.3%)	128 (66.7%)	36 (57.1%)
Medicare/Medicaid		85 (33.3%)	59 (30.7%)	26 (41.3%)
Personal Policy		5 (2.0%)	4 (2.1%)	1 (1.6%)
No Health Insurance		1 (0.4%)	1 (0.5%)	0 (0.0%)
**Employment**	n (%)			
Working		196 (76.9%)	149 (77.6%)	47 (74.6%)
Not working		59 (23.1%)	43 (22.4%)	16 (25.4%)
**Cohabitation status**	n (%)			
Living with partner		210 (82.4%)	168 (87.5%)	42 (66.7%)
Living without partner		45 (17.6%)	24 (12.5%)	21 (33.3%)
**Study year**	n (%)			
2017-2019		174 (68.2%)	138 (71.9%)	36 (57.1%)
2020-2022		81 (31.8%)	54 (28.1%)	27 (42.9%)
**24-hour time-use behaviors**				
MVPA[1] (min/day)	Geometric mean (%)	75.5 (5.2%)	72.6 (5.0%)	84.3 (5.9%)
LPA[2] (min/day)	Geometric mean (%)	311.2 (21.6%)	299.9 (20.8%)	344.0 (23.9%)
IA[3] (min/day)	Geometric mean (%)	593.9 (41.2%)	578.5 (40.2%)	636.0 (44.2%)

**Characteristic**	**Statistic**	**Overall** *N* = 255	**Sensitivity analysis** *N* = 192	**Excluded** *N* = 63
Sleep (min/day)	Geometric mean (%)	459.4 (31.9%)	488.9 (34.0%)	375.7 (26.1%)
**EPDS[4] score**	Median (IQR)	5.0 (2.0, 8.0)	5.0 (2.0, 8.0)	5.0 (3.0, 8.0)

[1]MVPA, moderate-to-vigorous physical activity; [2]LPA, light physical activity; [3]IA, inactivity; [4]EPDS, Edinburgh Postnatal Depression Scale.

References

1. Dumuid D, Stanford TE, Martin-Fernández JA, Pedišić Ž, Maher CA, Lewis LK, et al. Compositional data analysis for physical activity, sedentary time and sleep research. Stat Methods Med Res. 2018 Dec 1;27(12):3726–38. doi:10.1177/0962280217710835

2. Duan N. Smearing Estimate: A Nonparametric Retransformation Method. J Am Stat Assoc. 1983;78(383):605–10. doi:10.2307/2288126

3. Manning WG, Mullahy J. Estimating log models: to transform or not to transform? J Health Econ. 2001;20(4):461–94. doi:10.1016/s0167-6296(01)00086-8
